# Insulin Receptor Substrate-1 Associates with Small Nucleolar RNA Which Contributes to Ribosome Biogenesis

**DOI:** 10.3389/fendo.2014.00024

**Published:** 2014-03-04

**Authors:** Atsufumi Ozoe, Meri Sone, Toshiaki Fukushima, Naoyuki Kataoka, Kazuhiro Chida, Tomoichiro Asano, Fumihiko Hakuno, Shin-Ichiro Takahashi

**Affiliations:** ^1^Graduate School of Agriculture and Life Sciences, The University of Tokyo, Tokyo, Japan; ^2^Institute of Biomedical and Health Sciences, Hiroshima University, Hiroshima, Japan; ^3^Laboratory for Malignancy Control Research, Medical Innovation Center, Graduate School of Medicine, Kyoto University, Kyoto, Japan

**Keywords:** insulin-like growth factor, insulin receptor substrate, snoRNA, *U96A*, RACK1

## Abstract

Insulin receptor substrates (IRSs) are well known to play crucial roles in mediating intracellular signals of insulin-like growth factors (IGFs)/insulin. Previously, we showed that IRS-1 forms high molecular mass complexes containing RNAs. To identify RNAs in IRS-1 complexes, we performed ultraviolet (UV) cross-linking and immunoprecipitation analysis using HEK293 cells expressing FLAG–IRS-1 and FLAG–IRS-2. We detected the radioactive signals in the immunoprecipitates of FLAG–IRS-1 proportional to the UV irradiation, but not in the immunoprecipitates of FLAG–IRS-2, suggesting the direct contact of RNAs with IRS-1. RNAs cross-linked to IRS-1 were then amplified by RT-PCR, followed by sequence analysis. We isolated sequence tags attributed to 25 messenger RNAs and 8 non-coding RNAs, including small nucleolar RNAs (snoRNAs). We focused on the interaction of IRS-1 with *U96A* snoRNA (*U96A*) and its host Rack1 (receptor for activated C kinase 1) pre-mRNA. We confirmed the interaction of IRS-1 with *U96A*, and with RACK1 pre-mRNA by immunoprecipitation with IRS-1 followed by Northern blotting or RT-PCR analyses. Mature *U96A* in IRS-1^−/−^ mouse embryonic fibroblasts was quantitatively less than WT. We also found that a part of nuclear IRS-1 is localized in the Cajal body, a nuclear subcompartment where snoRNA mature. The unanticipated function of IRS-1 in snoRNA biogenesis highlights the potential of RNA-associated IRS-1 complex to open a new line of investigation to dissect the novel mechanisms regulating IGFs/insulin-mediated biological events.

## Introduction

Insulin-like growth factors (IGFs) and insulin display a variety of bioactivities, including embryonic development and growth, postnatal somatic growth, and regulation of glucose, lipid, and protein metabolism (1). These bioactivities have been shown to be accomplished by IGF/insulin signaling pathways composed of many signaling molecules (2–4). Insulin receptor substrates (IRS)-1 and IRS-2 are important substrates of the receptor-intrinsic tyrosine kinases and serve as adaptor proteins transmitting IGF/insulin signaling from the IGF-I/insulin receptor to the downstream effectors. Although the differences in the roles of these IRS isoforms in mediation of the IGF/insulin bioactivities remain largely unknown, it is suggested that IRS-1 plays a predominant role in somatic growth, and IRS-2 in glucose homeostasis, especially in liver (5–7). Following tyrosine phosphorylation of IRSs, Src homology region 2 (SH2) domain-containing proteins bind to IRSs leading to the activation of phosphatidylinositol 3-kinase (PI3K) and mitogen-activated protein kinase (MAPK) pathways. The activated PI3K transmits the signal to up-regulate growth and metabolism through the Akt signaling pathway (8). In particular, the downstream mammalian target of rapamycin (mTOR) is thought to play crucial roles in promoting growth-related intracellular activities such as ribosome biogenesis (9, 10). Since previous reports have shown that aberrant expression of IRS is linked to certain types of cancers (11–13), unraveling the signaling cascade of biological events that is involved in IRS regulation is important to understand the role of IRSs in human pathologies.

The eukaryotic ribosome is a large complex composed of four RNA molecules, including the 5S, 5.8S, 18S, and 28S ribosomal RNAs (rRNAs) (14) and about 80 distinct ribosomal proteins (RPs) (15). Ribosome biogenesis involves several coordinated steps, such as synthesis, post-transcriptional modification and processing of rRNA in the nucleus, synthesis of RPs and their import into the nucleus, the assembly of ribosome subunits, and the transport of the mature 40S and 60S subunits into the cytoplasm (16). The post-transcriptional modifications of rRNA include the two types of chemical modifications, pseudouridine and 2′-O-methylation, and are added by small nucleolar RNP complexes (snoRNPs) (17, 18) consisting of snoRNAs and several proteins. For both types of modification, site specificity is achieved by the snoRNA through base pairing with the target region. In vertebrates, the great majority of snoRNAs is encoded within introns of pre-mRNAs (19, 20) and they are processed from the debranched host introns (21–24). Therefore, the mature intronic snoRNA is produced concomitantly with splicing of the host pre-mRNA (22, 25).

Recently, we found that IRSs form high molecular mass complexes containing RNAs as well as a variety of proteins that modulate or mediate insulin-like bioactivities (29). In addition, we identified components of messenger ribonucleoprotein (mRNP) as IRS-1-associated proteins (30). Here, we present the results of the screening of RNA components of IRS-1 complex and found that IRS-1 can form a complex with snoRNA, *U96A*, and its host pre-mRNA, *Rack1* (*receptor for activated C kinase 1*) RNA. In this study, we propose the unanticipated role of IRS-1 in the biogenesis of *U96A*. This may be a novel mechanism to support the induction of IGF/insulin bioactivities.

## Materials and Methods

### Materials

Anti-IRS-1 antibody was raised in rabbits as described (31). The following peptide and antibodies were purchased: FLAG (F3165, Sigma, St. Louis, MO, USA), anti-FLAG antibody-conjugated agarose beads (A2220, Sigma), and anti-Myc antibody (9E10, Millipore, Billerica, MA, USA). All other chemicals were of reagent grade and were obtained commercially.

### Plasmids

To generate FLAG-tagged or Myc-tagged IRS-1 and IRS-2, the open reading frame of rat IRS-1 and human IRS-2 was subcloned into the pCMV–FLAG-2 vector (Sigma) or pCMV–Myc vector in-frame as described previously (32, 33). Full-length rat PABPC1 was subcloned into the pCMV mammalian expression vector containing an N-terminal FLAG tag (FLAG–PABPC1) as follows. An *Eco*RI restriction site was introduced at the 5′ end of the PABPC1 open reading frame and a *Sal*I site at the 3′- end by PCR using the oligonucleotides 5′-TTAAGAATTCAAGATGAACCCCAGCGCCCCCAGCTA-3′ and 5′-TTAAGTCGACTTAGACAGTTGGAACACCAGTGG-3′, respectively. The PCR product was digested with *Eco*RI and *Sal*I and subcloned into pCMV–FLAG cut with *Eco*RI and *Sal*I. Full-length human 15.5K was subcloned into the pCMV mammalian expression vector containing an N-terminal FLAG tag (FLAG-15.5K) or N-terminal GFP tag (GFP-15.5K) as follows. A *Bgl*II restriction site was introduced at the 5′ end of the 15.5K open reading frame and a *Sal*I site at the 3′ end by PCR using the oligonucleotides 5′-TTAAGAATTCAAGATGAACCCCAGCGCCCCCAGCTA-3′ and 5′-TTAAGTCGACTTAGACAGTTGGAACACCAGTGG-3′. The PCR product was digested with *Bgl*II and *Sal*I and subcloned into pCMV–FLAG and pEGFP-C1 cut with *Bgl*II and *Sal*I. Full-length human coilin was subcloned into the pCMV mammalian expression vector containing an N-terminal GFP tag (GFP–coilin) as follows. A *Kpn*I restriction site was introduced at the 5′ end of the coilin open reading frame and a *Bam*HI site at the 3′ end by PCR using the oligonucleotides 5′-TTAAGGTACCATGGCAGCTTCCGAGACGGTTAGGCTACG-3′ and 5′-TTAAGGATCCTCAGGCAGGTTCTGTACTTGATGTGTTACTTGG-3′. The PCR product was digested with *Kpn*I and *Bam*HI and subcloned into pEGFP-C1 cut with *Kpn*I and *Bam*HI.

Plasmids expressing Myc–IRS-1 fused with SV40 large T antigen-derived Nuclear Localizing Signal (NLS, PKKKRKV) in its C-terminus was constructed by PCR using pCMV–Myc–IRS-1 as template and the oligonucleotides 5′-AAGGCTGTCCTTGGGGGATCC-3′ and 5′-ATCGGGATCCCTATACCTTTCTCTTCTTTTTTGGTTGACGGTCCTCTGGTTG-3′. The PCR product amplified was digested with *Bam*HI and subcloned into pCMV–Myc–IRS-1 cut with the same restriction enzyme.

For the *in vitro* RNA synthesis, *U96A* snoRNA sequence was amplified by PCR using the oligonucleotides 5′-TTAATCTAGACCTGGTGATGACAGATGGCATTGTCAG-3′ and 5′-TTAACTGGAGTTCAGAATTGCAGGACATGTCCTCACTCC-3′. The primers were engineered to contain *Xba*I and *Xho*I sites to introduce these restriction sites at the 5′ and 3′ end of the PCR product, respectively. The PCR product was digested with *Xba*I and *Xho*I and subcloned into the downstream of T7 promoter of pBS-KS(+) cut with *Xba*I and *Xho*I, which is referred to as pBS-KS(+)-*U96A* snoRNA. U6 small nuclear RNA (snRNA) sequence was also amplified by the PCR using the oligonucleotides 5′-TTAATCTAGAGTGCTCGCTTCGGCAGCACATATACTAAAATTGG-3′ and 5′-TTAACTCGAGAAAATATGGAACGCTTCACGAATTTGCGTGTCATCC-3′, and subcloned into the downstream of T7 promoter of pBS-KS(+) cut with *Xba*I and *Xho*I.

### Animals

Insulin receptor substrates-1 knockout mice were kindly donated by Dr. Kadowaki and Dr. Ohsugi (Faculty of Medicine, The University of Tokyo). All animal experiments in this study were performed according to procedures approved by the Committee on Laboratory Animal Care, Graduate School of Agriculture and Life Sciences, The University of Tokyo.

### Cell culture and transfection

Human embryonic kidney 293 (HEK293) cells were kindly provided by Dr. Koichi Suzuki (National Institute of Infectious Diseases, Tokyo, Japan). MCF-7 human breast cancer cells (ATCC No. CRL8305) were a kind gift from Dr. Yoichi Hayakawa (Tokyo University of Science, Tokyo, Japan). HEK293, MCF-7 cells, and HeLa cells were maintained at 37°C in a humidified CO_2_-controlled atmosphere in Dulbecco’s modified Eagle’s medium (DMEM) supplemented with 10% fetal bovine serum (FBS), 0.1% NaHCO_3_, 50 IU/ml penicillin, 50 μg/ml streptomycin, 100 μg/ml kanamycin, and 0.5 μg/ml amphotericin B. For the serum-starvation, cells were starved for 16 h in DMEM supplemented with 0.1% bovine serum albumin (BSA).

Primary mouse embryonic fibroblasts (MEFs) were prepared from littermates according to the previous report (34) and kept in culture for 2–3 weeks, respectively. Briefly, MEFs were prepared from E14.5 embryos. Embryos were dissociated by 0.025% trypsin in 0.2% EDTA at 37°C for 10 min and then treated with DNaseI. After filtering fibroblasts with a 70 μm cell strainer, they were cultured in DMEM supplemented with 10% FBS, 100 μg/ml streptomycin, 100 U/ml penicillin, and 2 mM glutamine. MEFs were cultured at 9% CO_2_ at 37°C and experiments were performed at first passages (P1–P4).

For the plasmid transfection, the expression vectors were transfected into HEK293 cells using Lipofectamine 2000 (Invitrogen, Carlsbad, CA, USA), according to manufacturer’s protocol.

### Immunoprecipitation and immunoblotting

Immunoprecipitation and immunoblotting were performed as described elsewhere (30).

### Cross-linking and immunoprecipitation

Cross-linking and immunoprecipitation (CLIP) was performed using serum-starved HEK293 cell expressing FLAG–IRS-1 or FLAG–IRS-2 according to Ule et al. (35) with minor modification.

### Nucleus and cytoplasm fractionation

Nucleus/cytoplasm fractions were prepared according to previous report (36).

### RNA extraction, RT-PCR, and quantitative RT-PCR

Total RNA was extracted from cells using the TRIzol reagent (Invitrogen) followed by DNase I treatment. For RT-PCR, 1 μg of total RNA was reverse transcribed using random primers (Invitrogen) and SuperScript II reverse transcriptase (Invitrogen). A fraction of the RT reaction products was used in subsequent PCR reactions. Amplification parameters were denaturation at 94°C for 30 s, annealing at 64°C for 30 s, and extension at 72°C for 30 s in a 30-cycle program. Primer sequences were as follows: *Rack1 exon1–exon8* (5′-ACTGAGCAGATGACCCTTCGTG 3′ and 5′-GTTGTCCGTGTAGCCAGCAAAC-3′, 912 bp). The simultaneous analysis of serial dilution of the amount of RNA used in the RT-PCR reactions ensured that these reactions were quantitative.

For quantitative RT-PCR, the cDNA prepared was subjected to real-time PCR (ABI StepOne™ Real-Time PCR Systems), using SYBR Green Real-time PCR Master Mix Plus (TOYOBO, Osaka, Japan). Data were expressed as relative mRNA levels normalized to housekeeping gene (*Gapdh*) expression level in each sample. The primer sequences are as follows: *Rack1 exon2–intron2* (5′-GATGGTCAGTTTGCCCTCTC-3′, 5′-CTCAGTTCTGCCCACTTTCC-3′), *Rack1 exon3–exon4* (5′-GTCCCGAGACAAGACCATAAAG-3′, 5′-TGATAGGGTTGCTGCTGTTC-3′), *Gapdh* (5′-GTGTTCCTACCCCCAATGTG-3′, 5′-CCTGCTTCACCACCTTCTTG-3′).

### Northern-blot analysis

To detect small RNAs, total RNA was separated on a 10% denaturing acrylamide gel and transferred to Hybond-N^+^ membrane (GE Healthcare Biosciences, Pittsburgh, PA, USA) using a Trans-Blot SD Semi-Dry Transfer Cell (Bio-Rad, Hercules, CA, USA). For the detection of *U96A* snoRNA, DNA probes were synthesized with a Megaprime DNA labeling Kit (GE healthcare) and [α^−32^P]dCTP using *U96A* snoRNA cDNA as a template. The probes were hybridized using prehybridization buffer (50% deionized formamide, 5 × SSPE, 5 × Denhardt, 10% dextran sulfate, 0.1% SDS, 20 μg/ml salmon sperm) at 50°C. Densitometric analysis of RNA bands was performed using ImageJ gel analysis software (http://rsbweb.nih.gov/ij/).

### Co-immunoprecipitation of *U96A* snoRNA with proteins

HEK293 cells expressing FLAG-tagged proteins were serum-starved and washed twice with ice-cold PBS and irradiated with 4000 J/m^2^ ultraviolet (UV) in ice-cold PBS. The cells were collected in 1.5 ml microtubes and pelleted by centrifugation. The cell pellets were resuspended with 1 ml of NET-2 [50 mM Tris–HCl, pH 7.4, 300 mM NaCl, 0.05% NP-40, 500 μM Na_3_VO_4_, 10 μg/ml leupeptin, 5 μg/ml pepstatin, 20 μg/ml phenylmethylsulfonyl fluoride (PMSF); 100 KIU/ml aprotinin] containing 100 U of RNase-inhibitor (Ambion, Austin, TX, USA) and were incubated for 10 min on ice. Cells were lysed by 15 (3 × 5) bursts using a Branson Sonifier. Homogenates were centrifuged at 14,000 *g* for 10 min at 4°C, cleared by incubation with 20 μl Protein G-Sepharose beads (GE Healthcare) for 60 min, and incubated with a specific antibody for 1.5 h at 4°C, after which 30 μl Dynabeads protein G paramagnetic beads (Invitrogen) were added with gentle mixing for another 30 min at 4°C. The beads were washed five times with high-salt wash buffer [50 mM HEPES–KOH, pH 7.5, 500 mM KCl, 0.05% (*v*/*v*) NP-40, 0.5 mM DTT] and proteins on beads were eluted by incubation with lysis buffer containing 150 ng/ml FLAG peptide. Nine-tenth of the eluates was treated with Proteinase K, and the eluted RNAs were purified with the TRIzol Reagent and analyzed by Northern blotting. The remainder of the eluates was fractionated by SDS-PAGE and analyzed by immunoblotting.

### *In vitro* RNA synthesis

pBS-KS(+)-*U96A* snoRNA 0.5 μg linearized with *Xho*I and gel purified was used as a template for *in vitro* transcription with Riboprobe *in vitro* Transcription kit (Promega, Madison, WI, USA). The RNA products were treated with DNase I and purified with phenol/chloroform/isoamyl alcohol mixture and ethanol precipitation. The amount of transcribed RNA was quantitated by absorbance at 260 nm and the product size and purity were verified by 10% denaturing acrylamide gel.

### Gel shift assay

To obtain FLAG-tagged proteins, whole-cell lysates from HEK293T cells were prepared from transfected cells with lysis buffer (20 mM HEPES, pH7.5, 150 mM NaCl, 1% Triton X-100, 1 mM DTT, 1 mM EDTA, 10% glycerol, 10 μg/ml leupeptin, 5 μg/ml pepstatin, 20 μg/ml PMSF, 100 KIU/ml aprotinin). FLAG-tagged proteins were purified from whole-cell lysates by using anti-FLAG-M2 affinity resin, washed with high-salt buffer (20 mM HEPES, pH7.5, 400 mM NaCl, 1% Triton X-100, 1 mM DTT, 1 mM EDTA, 10% glycerol, 10 μg/ml leupeptin, 5 μg/ml pepstatin, 20 μg/ml PMSF, and 100 KIU/ml aprotinin), and eluted with FLAG peptide. Purity of the eluted proteins was verified by SDS-PAGE, followed by Coomassie Brilliant Blue staining.

Gel shift assay was conducted using ~50,000 cpm *in vitro* transcribed, [^32^P]-UTP-labeled *U96A* snoRNA (~100 nt), *U95* snoRNA (~100 nt), or U6 snRNA probe (~120 nt). The probe was heat denatured for 5 min by heating at 65°C and renatured prior to being added to binding reactions containing FLAG–GFP, FLAG–IRS-1, FLAG–15.5K proteins (100 ng). The binding reaction was performed at 4°C for 45 min in 20 μl binding buffer (20 mM HEPES-KOH, pH7.5, 6 mM MgCl_2_, 60 mM KCl, 7.5% glycerol, 4 mM DTT, 125 ng/μl yeast tRNA, 50 ng/μl BSA, and 5 U RNase-inhibitor) was added. Bound complexes were resolved on native 5% polyacrylamide gels in 0.25 × TAE buffer. Autoradiography was performed using a FLA-5000 imaging system (Fujifilm Life Sciences, Tokyo, Japan).

### Measurement of DNA contents

DNA content in cells was determined using PicoGreen dsDNA Quantitation Reagent (Invitrogen) according to manufacturer’s procedure.

### FACS analysis

For the analysis of cell cycle distribution, the cells were starved for 48 h in DMEM plus 0.1% FBS. MEFs were trypsinized and thoroughly dissociated into single cells in FACS buffer [0.1% FBS, 1mM EDTA, 1 mM NaCl in PBS(−)] and passed through a 40 μm cell strainer (BD Falcon). The collected cells were fixed in 70% ice-cold ethanol overnight at −20°C. Fixed cells were subsequently stained with 20 g/ml Propidium Iodide diluted in PBS containing 0.2 mg/ml RNAse A for 30 min at 37°C. Cells were sorted and counted using BD FACSCalibur (BD Biosciences, San Jose, CA, USA) according to the manufacturer’s instruction. All the analyses were performed at least three times on different genetic backgrounds.

### Immunofluorescence

HeLa cells and MCF-7 cells grown on coverslips were serum-starved for 16 h. The cells were fixed in 4% paraformaldehyde/PBS for 15 min at room temperature, permeabilized with PBS containing 0.25% Triton X-100 for 5 min at room temperature, blocked with blocking buffer (3% BSA and 0.025% NaN3 in PBS) for 1 h at room temperature. Then, primary antibody against Myc epitope was added and incubated overnight at 4°C. After the samples were washed with PBS, secondary antibody incubation was done for 1 h at room temperature using anti-mouse Alexa Fluor 594. The slides were mounted with Vectashield mounting medium (Vector Laboratories, Burlingame, CA, USA). Images were captured using FV500 confocal microscope (Olympus, Tokyo, Japan) and analyzed with Fluoview version 1.4 and Photoshop CS5.

### Statistical analysis

Data are expressed as means ± standard error of mean (SEM). Comparisons between two groups were performed using Student’s *t*-test.

## Results

### IRS-1, but not IRS-2 forms complexes with RNAs in cells

**Figure 1:**
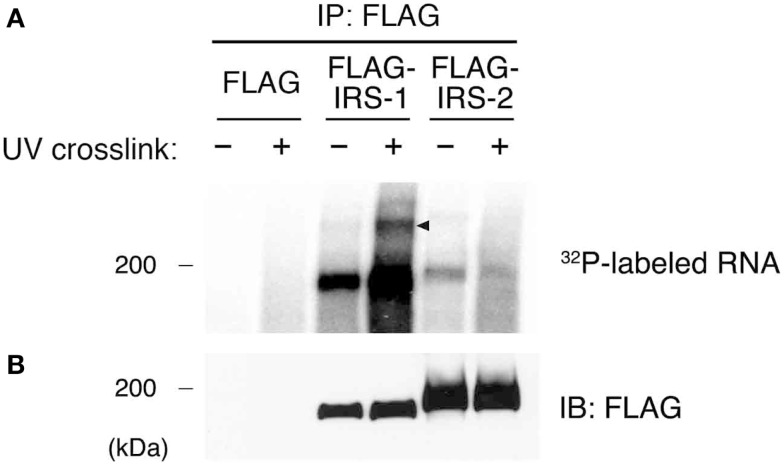
**Purification of IRS-1-RNA complexes by CLIP**. **(A)** HEK293 cells expressing FLAG–IRS-1 or IRS-2 were irradiated with or without UV. Protein–RNA complexes were immunoprecipitated with anti-FLAG antibody, and the 5′ ends of immunoprecipitated RNAs were radiolabeled with ^32^P. Proteins cross-linked with radiolabeled RNAs were separated by SDS-PAGE and visualized by autoradiography. Representative autoradiograms from three experiments are shown. RNA–protein complexes of ~200 kDa (arrowhead) are seen with FLAG–IRS-1 immunoprecipitates (IP) dependent on UV irradiation but not control and FLAG–IRS-2 IP. **(B)** Immunoblot (IB) analysis of IRS-1 and IRS-2 IP using anti-FLAG antibody.

Our previous studies showed that IRS-1 forms high molecular mass complexes with mRNPs (30). Importantly, IRS-1 but not IRS-2 was incorporated into mRNPs (30). Prior to identifying RNAs in IRS-1 complex, we first examined whether IRS-1 interacts with RNAs in close proximity or not. To this end, we performed an UV CLIP analysis (35, 37). HEK293 cells expressing FLAG–IRS-1 or FLAG–IRS-2 were irradiated with UV to cross-link between proteins and RNAs in living cells. Cells were lysed, and RNAs were partially cleaved by the treatment with low-concentrations of RNase, providing short RNA tags covalently cross-linked to target proteins. FLAG–IRSs were then immunoprecipitated and 5′ ends of cleaved RNAs in the precipitates were radiolabeled with polynucleotide kinase (PNK) and [γ^−32^P]ATP. The immunoprecipitates were then separated by SDS-PAGE, transferred onto nitrocellulose membrane, and detected by autoradiography and immunoblotting (Figures 1A,B). In samples without UV irradiation, we observed radioactive signals at 180 kDa around the band of IRS-1, indicating non-specific phosphorylation of IRS-1 by PNK *in vitro*. However, UV irradiation greatly increased the radioactive signals at 180 kDa, indicating that IRS-1 covalently interacted with RNAs. In the CLIP analysis, a portion of radioactive signals of the protein–RNA complexes showing slower electrophoretic mobility are often observed because of covalent interaction with longer RNAs (35). Indeed, we actually observed weak but specific radioactive signals with slower electrophoretic mobility than that of IRS-1 itself (Figure 1A). In contrast to IRS-1, UV irradiation did not increase the radioactive signals around the band of IRS-2 (Figure 1A) suggesting that IRS-2 does not interact with RNAs. These results clearly indicated that IRS-1 is in direct contact with RNAs in cells.

### IRS-1 complexes contain *U96A* snoRNA

We next sought to identify RNAs that are in direct contact with IRS-1 in cells. For this purpose, we extracted RNAs from the radioactive region around IRS-1–RNA complexes (Figure 1, *arrowhead*) and amplified them by RT-PCR, followed by sequence analysis. We excluded tags with imperfect (<80%) matches to genomic sequences, and accordingly identified 33 IRS-1-bound RNAs from three experiments, which had an average length of 103 nucleotides (Tables A1 and A2 in Appendix). The resulting set of tags included not only tags mapped to 25 messenger-RNA-encoding genes (Table A1 in Appendix) but also tags mapped to 8 non-coding RNAs including snoRNAs and snRNAs (Table A2 in Appendix). RNA sequences that we determined were mapped not only to exons but also to introns or 3′UTRs. As negative control experiments, we repeated CLIP analysis using cells expressing FLAG, but could not amplify the tags.

**Figure 2:**
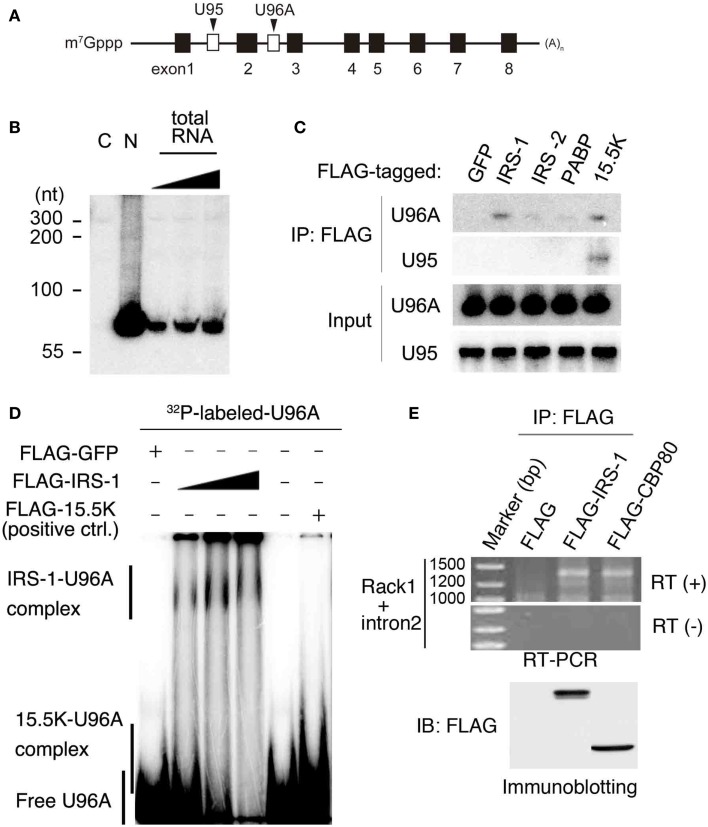
**Complex formation of IRS-1 with *U96A* snoRNA**. **(A)** *U96A* is located in the second intron of human *Rack1* gene. Human *Rack1* gene contains 8 exons (black boxes) and 2 snoRNAs (white boxes). *Rack1* produces mature *Rack1* mRNA and two non-coding RNAs, *U95* and *U96A*. **(B)** MCF-7 cells were separated into cytosolic (C) and nuclear (N) fractions. RNA was prepared from both fractions and 10 μg RNAs from each fraction were analyzed for *U96A* snoRNA abundance by Northern-blot analysis. 100, 33, and 11% of 10 μg total RNAs were also loaded. Representative results from at least four independent experiments are shown. **(C)** Lysates from HEK 293 cells overexpressing FLAG–IRS-1, IRS-2, PABPC1 (negative control) or 15.5 K (positive control) were subjected to immunoprecipitation with anti-FLAG antibody. RNA–protein complexes were eluted with 3 × FLAG peptides, and RNAs bound to the proteins were extracted with phenol/chloroform/isoamyl alcohol mixture and ethanol precipitation. RNAs were subjected to Northern blotting with the indicated probes. Representative results from three independent experiments are shown. **(D)** Gel shift assay was conducted using *in vitro* transcribed, [^32^P]-UTP-labeled *U96A* probe. The *U96A* probe was added to binding reactions containing FLAG–IRS-1 proteins (50, 100, 200 ng), FLAG-tagged GFP (negative control) or FLAG-15.5K (positive control). Representative results from three independent experiments are shown. **(E)** Lysates from HEK 293 cells overexpressing FLAG, FLAG–IRS-1, FLAG–CBP80 were subjected to immunoprecipitation with anti-FLAG antibody. RNA–protein complexes were eluted with 3 × FLAG peptides, and RNA bound to the proteins was extracted with phenol/chloroform/isoamyl alcohol mixture and ethanol precipitation. RNAs were subjected to RT-PCR using the primers specific for *Rack1*, which are designed within the first and last exon. Representative results from three independent experiments are shown.

Among IRS-1 bound RNAs, we focused on *U96A snoRNA* (*U96A*), a member of the Box C/D type snoRNAs, which are well established to function in post-transcriptional chemical modification of rRNA (19). *U96A* is encoded in the second intron of the *Rack1* gene (Figure 2A) (38). We first examined the expression of *U96A* in MCF-7 cells by Northern-blot analysis and the result showed that *U96A* was expressed and localized in the nucleus (Figure 2B), which is the same subcellular distribution as canonical box C/D snoRNAs. The interaction of IRS-1 with *U96A* was confirmed by co-immunoprecipitation assay, followed by Northern-blot analysis using labeled *U96A* as a probe (Figure 2C). We neither detected U96A in the immunoprecipitates of GFP, IRS-2 nor poly-A binding protein (PABP). In a positive control experiment, U96A was detected in the RNAs co-purified with 15.5K, which is known to primarily interact with Box C/D type snoRNAs as a component of snoRNP (19, 22, 39). Interestingly, *U95* snoRNA, which is encoded in the first intron of *Rack1* pre-mRNA was not co-immunoprecipitated with IRS-1. The direct interaction between IRS-1 and *U96A* was also confirmed by *in vitro* binding assay (Figure 2D).

Because snoRNAs were generally excised from an intron of protein-coding mRNAs coupled with splicing (22, 25), we examined the possibility that IRS-1 also interacts with *Rack1* pre-mRNA. RNAs co-immunoprecipitated IRS-1 was subjected to RT-PCR using *Rack1* specific primers designed within the first and last exon. As a result, we detected single band with a size around 1400 bases (Figure 2E). By sequencing analysis, The PCR product was proved to be *Rack1* pre-mRNA that retains only intron 2. A similar size DNA was amplified using RNAs co-immunoprecipitated with CBP80, a protein that can bind pre-mRNAs (40, 41).

### IRS-1 positively regulates *U96A* snoRNA biogenesis

**Figure 3:**
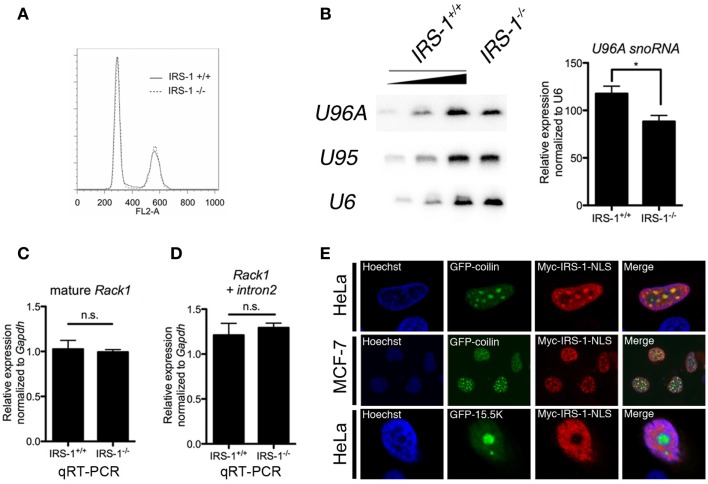
**Effects of IRS-1 on expression of U96A snoRNA**. Embryonic fibroblasts prepared from IRS-1^+/+^ and IRS-1^−/−^ mice were serum-starved for 48 h. Cells were harvested and split into three portions. One portion was subjected to DNA extraction, which was used to normalize the amount of RNA. Another portion was used as a source of RNA, which was analyzed by Northern blotting **(B)**, and by RT-PCR **(C,D)**. The remaining portion was used for cell cycle analysis **(A)**. **(A)** FACS analysis was performed to analyze the cell cycle distribution (*n* = 3). **(B)** Relative expression of U96A in MEFs from IRS-1^+/+^ and IRS-1^−/−^ animals. 100, 33, and 11% of IRS-1^+/+^ samples were loaded. *n* = 3, bars indicate SEM. *P < 0.05 (Student’s *t*-test). **(C,D)** Relative expression of mature Rack1 mRNA and Rack1 pre-mRNA retaining intron2 in MEFs from IRS-1^+/+^ and IRS-1^−/−^ animals. *n* = 5, bars indicate SEM. n.s., non-significant (Student’s *t*-test). **(E)** HeLa cells and MDF-7 cells were co-transfected with the plasmids expressing Myc–IRS-1 and either GFP–coilin (Cajal body marker) or GFP-15.5K (nucleolus marker). Following serum-starvation, cells were fixed and stained with anti-Myc antibody and Hoechist33342. A merge of the two images is shown with yellow indicating areas of colocalization. Representative stainings from at least three experiments using each cell are shown.

To gain insight into the biological significance of the association of IRS-1 with *Rack1* pre-mRNA and *U96A*, we examined the amount of *U96A* in embryonic fibroblasts derived from IRS-1-deficient mice (IRS-1^−/−^ MEFs) and wild-type MEFs (IRS-1^+/+^ MEFs). Embryonic fibroblasts prepared from littermate IRS-1^+/+^ MEFs and IRS-1^−/−^ MEFs were cultured under serum-free conditions and total RNAs were normalized to DNA content since cell cycle distribution was the same in IRS-1^+/+^ and IRS-1^−/−^ MEFs (Figure 3A). Northern-blot analysis showed that the *U96A* levels in IRS-1^−/−^ MEFs were less than those in WT MEFs (Figure 3B). These results demonstrated that IRS-1 positively regulates the abundance of *U96A*. It is also important to note that the amounts of both *Rack1* mature mRNA and *Rack1* pre-mRNA retaining only intron 2 were unchanged, suggesting that the effect of IRS-1 depletion on the reduction of *U96A* in IRS-1^−/−^ MEFs is not due to altered *Rack1* transcription and splicing (Figures 3C,D).

Small nucleolar RNAs are generally produced concomitantly with transcription/splicing of pre-mRNA in nucleoplasm and translocated to the nucleolus, which is the site of rRNA transcription and processing. Several snoRNAs are also reported to be detected in Cajal bodies, where they mature (20, 42). On the other hand, a part of IRS-1 is reported to be localized in nucleus (43–49). To investigate the possibility that nuclear IRS-1 is linked to snoRNA biogenesis, we performed immunofluorescent staining of NLS-fused Myc–IRS-1 (Myc–IRS-1–NLS) using HeLa cells and MCF-7 cells expressing either GFP–coilin or GFP-15.5K, a molecular component of Cajal bodies and nucleolar. As shown in Figure 3E, Myc–IRS-1–NLS certainly localizes to nucleus and exhibits a punctate staining pattern as well as general staining of the nucleoplasm. We found that Myc–IRS-1–NLS significantly colocalizes with GFP–coilin, suggesting that a function of IRS-1 in the nucleus might be related to snoRNA biogenesis.

## Discussion

We had shown that IRS-1 and RNAs co-exist in high molecular weight complexes in cells. In this study, we identified several RNAs that physically interact with IRS-1. They included some mRNA species and non-coding RNAs. Among them, we focused on U96A snoRNA, and our results suggest a novel role of IRS-1 in U96A biogenesis.

Using CLIP analysis, we demonstrated that IRS-1 is in close proximity to RNAs including some mRNA species and non-coding RNAs. *In vitro* binding assay using purified IRS-1 and U96A, one of RNAs associating with IRS-1, also suggest that they interact with each other directly, although it cannot be ruled out that another RNA-binding protein(s) is co-purified with IRS-1 and mediates the interaction of IRS-1 with U96A. Because IRS-1 does not possess a known RNA-binding domain as far as we searched, it is important to determine the region in IRS-1 responsible for interaction with RNAs.

Since snoRNAs are encoded in protein-coding genes (host genes), the expression level/efficiency of snoRNA is dependent on the transcription of their host genes and splicing (21, 22). We observed that the *U96A* levels were reduced in IRS-1^−/−^ MEF (Figure 3A), while the amounts of mature and intron2-retained pre-mRNA of *Rack1* were not affected (Figure 3C), indicating that IRS-1 positively regulates the *U96A* levels after the step in which *U96A* was removed from *Rack1* pre-mRNA by splicing. These steps include assembly of snoRNPs, debranching of removed lariat intron, or translocation of snoRNP into nucleolus.

We found that *Rack1* pre-mRNA that retains the only intron 2 was accumulated in the IRS-1-immunoprecipitates. We found that the second intron of *Rack1* where *U96A* is encoded possesses an atypical 5′ splice site (5′ss) with a GC dinucleotide in the first two intron positions, whose splicing-promoting activity is relatively weak. Thus, the retention of only the second intron may be due to this unique 5′ss sequence rather than the binding of IRS-1 to the intron. The mechanism by which this intrinsically weak 5′ss is efficiently selected by splicing factors and the GC 5′ ss-containing intron removal is promoted are poorly understood (50, 51). It is possible that the retention of the intron 2 is the rate-limiting step and plays important roles in expression of *U96A*. Given that our result shows IRS-1 forms a complex with *U96A* but not *U95* (Figure 2C), which is encoded within the first intron of *Rack1*, splicing retention of intron 2 may provide the temporal opportunity for IRS-1 to access to the site of encoding *U96A*.

It is well established that snoRNAs form snoRNP complexes, and function to guide modification enzymes to newly synthesized rRNAs in the nucleolus (52, 53). Thus, snoRNAs are essential for the post-transcriptional chemical modification of rRNA, which is required for ribosome maturation and functions such as rRNA processing, ribosome structure, and IRES-mediated translation (53–56). Although the loss of all of snoRNAs individually in yeast causes no obvious effect on cell growth (57), depletion of the modifications in multiple sites resulted in severe growth retardation (53, 58). Furthermore, recent studies have shown that dysfunction of snoRNAs and their host pre-mRNA may have a role in the human cancers (26), implying their contribution to the cancer development caused by the alterations of ribosome biogenesis (27, 28). On the other hand, although IRS-1 is predominantly a cytoplasmic protein, earlier studies demonstrated that endogenous IRS-1 is also detectable in the nucleus (43–49), where its precise functions and biological relevance remain largely unknown. So far, in the nucleus, it has been reported that IRS-1 regulates transcription of rRNA and several genes implicated in cell proliferation and cancer progression (47, 48). Our findings that repression of *U96A* in IRS-1 knockout cells and the presence of nucleus-targeted IRS-1 in Cajal bodies suggest that IRS-1 functions in ribosome biogenesis by affecting the quality control of rRNA as well as rRNA abundance, which may contribute to the efficient cell growth mediated by IRS-1 and cancer development as well.

**Figure 4:**
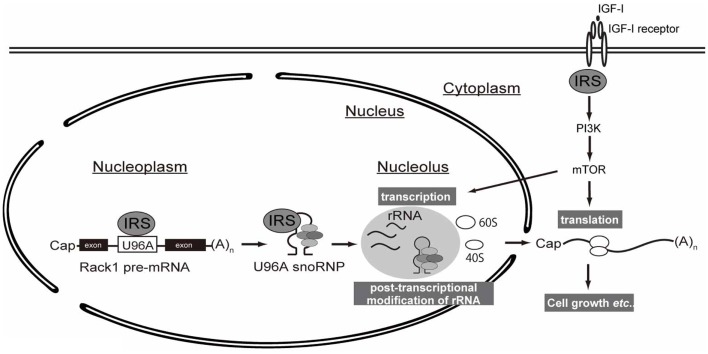
**A working hypothesis of IRS-mediated control of protein synthesis**. Following IGF stimulation, intracellular signaling through IRS activates downstream PI3K–mTOR signaling pathway, which is well known to promote translation of mRNAs. Up-regulation of protein synthesis is supported by ribosome maturation, which is accomplished by multiple coordinated processes, including rRNA transcription and ribosome activation (e.g., ribosomal protein S6 phosphorylation). The transcribed rRNAs in nucleus undergo chemical modification by snoRNP complexes whose biogenesis is controlled by nuclear IRS-1.

As explained in the introduction, IGF/insulin signals through IRSs induce translation of mRNA by activating the PI3K and MAPK pathways. In this study, we propose a novel mechanism by which IRSs could potentiate protein synthesis through the interaction of IRS-1 with snoRNA (Figure 4).

## Author Contributions

Atsufumi Ozoe, Toshiaki Fukushima, Naoyuki Kataoka, Kazuhiro Chida, Tomoichiro Asano, Fumihiko Hakuno, and Shin-Ichiro Takahashi conceived and designed the experiments. Atsufumi Ozoe, Meri Sone, and Toshiaki Fukushima performed the experiments. Atsufumi Ozoe, Meri Sone, Toshiaki Fukushima, Naoyuki Kataoka, Kazuhiro Chida, Fumihiko Hakuno, and Shin-Ichiro Takahashi analyzed the data. Atsufumi Ozoe, Meri Sone, Toshiaki Fukushima, Naoyuki Kataoka, Fumihiko Hakuno, and Shin-Ichiro Takahashi contributed reagents/materials/analysis tools. Atsufumi Ozoe, Naoyuki Kataoka, Fumihiko Hakuno, and Shin-Ichiro Takahashi wrote the paper. Shin-Ichiro Takahashi took the primary responsibility for final content. Atsufumi Ozoe, Meri Sone, Toshiaki Fukushima, Naoyuki Kataoka, Kazuhiro Chida, Tomoichiro Asano, Fumihiko Hakuno, and Shin-Ichiro Takahashi read and approved the final manuscript.

## Conflict of Interest Statement

The authors declare that the research was conducted in the absence of any commercial or financial relationships that could be construed as a potential conflict of interest.

## Appendix

**Table A1:** **Annotated list of IRS-1 CLIP tags (mRNA)**.

CLIP tag	Gene	Length	Location
GGTCACGATCTCACCCAGCTTAAGCTTGTAGGATCGTGGTCTTCCCT	ADP-ribosylation factor 1 (ARF1)	47	Exon 3
ATACCACCCTGAACGCGCCCGATCTCCTCTGATCTTTGAAGCTAAGCAGGGTCGGGGCTGGTTACTACTTGGATGGGAGACCGCCTGGGAATACCGGGTGCTGTGGGCTT	Hypothetical LOC728649	110	
ATCGACTGTGGGGGCGGCTATGTGAAGCTGTTTCCTATAGTTTGGACCAGACAGACATGCACGGAGACTCAGAATAC	Calreticulin (CALR)	77	Exon 3
GTTTCCACATACAAATTATAAAACGTGTTTATTTTGCTGGGCGCAGTGGCTCATGCCTGTAATCCTAGCACTTTGGGAGGCCAAGGCGGGTGGATCACCTGAGGTCAAGAGTTCGAGACCATCCTGGCCAACA	Family with sequence similarity 118, member A (FAM118A)	134	3’ UTR
GAGCCTGTCACCACGCCTGGCTAATTTTTTGTATTTTTAGTAGAGACGGGGTTTCACCACCACGTTGGCCAGGCTGGTCTCGAACTCTTGACCTCAGGGGATCCACCCACCTCGGCCTCCTAAAGTGCTGGGATTACGGGCGTCAGTCACCGCACCCGGCCCAAGACAGGTGATCTTTTTAAAGATCAACGTTGGT	IWS1 homolog (*S. cerevisiae*) (IWS1)	196	Intron
AGGACACATTGATCATCGACACTTCGAACGCACTTGCGGCCCCGGGTTCCTCCCGGGGCTACGCCTGTCTGAGCGTCGCT	Hypothetical protein LOC100132755	80	
AGTATTTGCTCCAGCTGATATTTTGGTAATATTTTCTAAAGATAAGAAAAGACAGGGGTTTCATATAAC	PABP1-dependent poly A-specific ribonuclease subunit (PAN3)	69	Intron
AGTCTATAGTCCTTGTGTGTATGGGTGGCACCATGATGGAGTGGAAACAGCACTGGCCT	Leucine rich repeat containing 1	59	Intron
AGCTGTTGAAGCTCAAAGCTGGAGGTGAGCTTCTGAGGCCTTTGCCATTATCCAGCCCAAGATTTGGTGCCTGCAGCCTCTTGTCTGGTTGAGGACTTGGGGCAGGAAAGGAATGCTGCTGAACTT	ATP-binding cassette, sub-family F (GCN20), member 1 (ABCF1)	126	3’ UTR
AACTCTTCCCAGATGATGACAATGAAATTAGTGCCTGTTTTCTTGCAAATTTAGCACTTGGAAC	TIMP metallopeptidase inhibitor 3 (Sorsby fundus dystrophy, pseudoinflammatory) (TIMP3)	64	3’ UTR
AACCGGGTGACCGAGATCCTCGATT	Component of oligomeric Golgi complex 4 (COG4)	25	Exon19
TCTGCCACTAATCAATCCTTCTGTATTTTCCTTTGACCCTGTTCACTGGGGGTGGTGGGCAGAGATCCAAACTCTTTAATTCTGCATCGTCCTCTCT	Syntaxin 7 (STX7)	97	Exon4/5 junction
AATCCATCCTCGGATGTCGCTGCCTTGCATAAGGCCATAATGGTTAAAGGTGTGGATGAAGCAACCATCATTGACATTCTAACTAAGCGAAAC	Annexin A1 (ANXA1)	93	Exon2/3 junction
AAGGAGAAGCGGGTAGGAGGTGTGCATGGCACCTCCGTCAGTCTTTGCCGAGGTTCCGCAGGCCCAGCCTGTCCTGGTCTTCAAGCTCACTGCCGACTTCAGGGAGGATCCGGACCCCCGCAAGGTCAACCTGGGAGTGGGAGCATATCGCACGGATGACTGCCATCCCTGGGTTTTGCCAGTAGTGAAGAAAGTGGAGCAGAAGATTGCTAATGACAATAGCCTAAATCACGAGTATCTGCCAATCCTGGGCCTGGCTGAGTTCCGGAGCTGTGCTTCTCGTCTTGCCCTTGGGGATGACAGCCCAGC	Glutamic-*o*xaloacetic transaminase 1, soluble (aspartate aminotransferase 1) (GOT1)	309	Exon1/2 junction
TATCAAGCTAGCCAGGCTCTTAACTGCTGTGTTGATGAAGAACATGGAAAAGGGTCCCTAGAAGAAGCTGAAGCAGAAAGACTTCTTCTAATTGCAAGTAAGTGTGATGCACCTGAAAGAGTTCCAACGTGTCAG	Anillin (ANLN)	135	Exon13
AGTCTTCGTTCCACTGGACAGGGAAAGCTTGAAACTTTGGCTCTGCCGTCCAGAAAGGTTTGTTTTCAGAAGCACTTCCTTTTCC	SMEK homolog 1, suppressor of mek1 (Dictyostelium) (SMEK1)	85	Exon15
GAAACAAACAGTTCTGAGACCGTTCTTCCACCACTGATTAAGAGTGGGGTGGCAGGTATTAGGGATAATATTCATTTAGCCTTCTGAGCTTTCTGGGCAGACTTGGTGACCTTGCCAGCTCCAGCAGCCTTCTTGTCCACTGCTTTGATGACACCCACCGCAACTGTCTGTCTCATATCACGAACAGCAAAGCGACCCAAAGGTGGATAGTCTGAGAAGCTCTCAA	Eukaryotic translation elongation factor 1 alpha 1 (EEF1A1)	226	Exon8
CCTCATCAAGAAAGGTTTGTTTATAGTATTTTTACTATAGCTTCATCCTTGATAACGTCCTAATTTCCTTCTGGACAACCTCCTTGACTAATGGCATATTGAGATCTATGTGACATGAGGATATTTCTCAGTACCACTTTGTTACTGGTACCTGATGCACACGGATTGCCACCAGAGCATGATGCCTCCATCAAGTGGTAATATGTTTGCAGCCTGCTGTCCAGCCAAGA	Zinc finger protein 317 (ZNF317)	230	3’ UTR
AAGCGTTGTTAGGTTTTTGTGTAAGATTCTTGCTGTAGCGTGGATAGCTGTGATTGGTGAGTCAACCGTCTGTGGCTACCAGTTACACTGAGATTGTAAC	Acidic (leucine-rich) nuclear phosphoprotein 32 family, member B (ANP32B)	100	3’ UTR
ATTTTCAGAGTCTTGTCCCCGGGGTATTAGCACCTCTTTTTGAACAGGGAATTGATTCAAGATTGGACATGGTCTCCTCT	Citrate synthase (CS)	80	3’ UTR
GTTCTCCAGGATGGCGCAGATGGTACGGGTAGTGGCGCACAT	Seryl-tRNA synthetase (SARS)	42	Exon 10
AAGCGGCAATTTCCAGTTTCTATTCGTGGAACTGGAATGCCATAGATGAGGGGCCCAAGAGGGACATTGTCAAGGAACCTGAGGTAGCCATTAGGAACAGAACTGACCTGCGT	Fucosidase, alpha-l-2, plasma (FUCA2)	113	Exon3
CTGACACGAGTGTCAGTGATGGTGAGGTAAGTTCT	Ribosomal RNA processing 12 homolog (*S. cerevisiae*) (RRP12)	35	Exon18
GAGTGTATAACGGTATACAACAAAATACTTTTTGTAATTAAATCTAGTCACTTTGACTCTAAGTAAATTCT	Homo sapiens similar to FUS interacting protein (serine–arginine rich) 1	71	3’ UTR
ACAAAGTTTGAGAATGCTTTCTTGTCTCATGTCGTCAGCCAGCACCAAGCC	ATP synthase, H + transporting, mitochondrial F1 complex, alpha subunit 1, cardiac muscle (ATP5A1)	51	Exon12

**Table A2:** **Annotated list of IRS-1 CLIP tags (non-coding RNA)**.

CLIP tag	Gene	Length	Location
ACAGCCGCGCTGAGAATGAGCCCCGTGTGGTTGGTGCGCGGACACGCACTGCCTGCGTAACTAGAGGGAGCTGACGGATGACGCCCCCGCGCCACGCCGCTCAGCGGGATACGCTTCTT	RNA component of mitochondrial RNA processing endoribonuclease (RMRP)	119	
GCCGCCATCTTTTCCTGTGTGACCGCACATGTCCACCACCATGCTAACCACTTAACAAAGCCTCGCCCATCATGATGTGGCCTTCCCGCCACTTGAACACTGCGACAGAACTGGATCCGCCATTTTTCCTCCA	X (inactive)-specific transcript (XIST)	133	
AGAAGATTAGCATGGCCCCTGCGCAAGGATGACACGCAAATTCGTGAAGCGTTCCATATTTTT	U6 small nuclear RNA (U6 snRNA)	63	
CAGTTTAATATCTGATACGTCCTCTATCCGAGGACAAT	U2 small nuclear RNA (U2 snRNA)	38	
CCTGGTGATGACAGATGGCATTGTCAGCCAATCCCCAAGTGGGAGTGAGGACATGTCCTGCAATTCTGAAGGG	C/D box 96A small nucleolar RNA (SNORD96A)	73	
ACCACTCAGACCGCGTTCTCTCCCTCTCACTCCCCAATACGGAGAGAAGAACGATCATCAATGGCTGACGGCAGTTGCAGCCAAGCAACGCCAGAAAGCCGGCTTCACGCTCAGGAGAAAACGCTACCTCTCTTCCTCGTGGTTTTCGGTGCTCTACACGTTCAGAGAAACTTCTCTAGTAACAC	C/D box 3A small nucleolar RNA (SNORD3A)	185	
ACCGAAAACCAAGAGGAAGAGAGGTAGCGTTTTCTCCTGAGCGTGAAGCCGGCTTTCTGGCGTTGCTTGGCTGCAACTGCCGTCAGCCATTGATGATCGTTCTTCTCTCC	C/D box 3A small nucleolar RNA (SNORD3A)	110	
TTGGTCAGACGGGTAATGTGCCTACGTCGTAACAAGGTTC	C/D box 13 small nucleolar RNA (SNORD13)	40	

## Abbreviations

CLIP, UV cross-linking and immunoprecipitation; DMEM, Dulbecco’s modified Eagle’s medium; FBS, fetal bovine serum; HEK293, human embryonic kidney 293; IGFs, insulin-like growth factors; IRSs, insulin receptor substrates; MAPK, mitogen-activated protein kinase; mRNA, messenger RNA; mRNP, messenger ribonucleoprotein; mTOR, mammalian target of rapamycin; PI3K, phosphatidylinositol 3-kinase; RNase A, ribonuclease A; snoRNA, small nucleolar RNA.
